# Human milk glycosaminoglycans: the state of the art and future perspectives

**DOI:** 10.1186/1824-7288-39-2

**Published:** 2013-01-15

**Authors:** Giovanni Valentino Coppa, Orazio Gabrielli, Enrico Bertino, Lucia Zampini, Tiziana Galeazzi, Lucia Padella, Lucia Santoro, Rita Lucia Marchesiello, Fabio Galeotti, Francesca Maccari, Nicola Volpi

**Affiliations:** 1 Pediatric Division, Department of Clinical Sciences, Polytechnic University of Marche, Ospedali Riuniti, Presidio Salesi, Via Toti 4, Ancona, 60121, Italy; 2 Department of Biology, University of Modena & Reggio Emilia, Modena, Italy; 3 Neonatal Intensive Care Unit, Department of Pediatrics, Faculty of Medicine and Surgey, University of Turin, Turin, Italy

## Abstract

Recently, a complete characterization and detailed evaluation of the glycosaminoglycans of human milk were performed. The total glycosaminoglycans content in milk from healthy mothers having delivered term or preterm newborns showed a constant pattern which was essentially composed of two main polysaccharides: chondroitin sulfate (60-70%) and heparin (30-40%). Moreover, considerable variations of glycosaminoglycans concentration were found during the first month of lactation, the highest values being present in colostrum compared to mature milk. Metabolism and potential biological functions of human milk glycosaminoglycans are hypothesized and future studies are encouraged.

## Background

In the last few decades consistent evidence has been reported on several human milk glycans (such as glycoproteins, glycolipids and especially oligosaccharides) which have been demonstrated to possess specific biological properties, positively influencing the breastfed newborn health [1-4]. On the contrary, only four studies are actually available in the literature on another family of complex carbohydrates, the glycosaminoglycans (GAGs) [5-8].

GAGs are linear heteropolysaccharides composed of a variable number of repeating disaccharidic units, which are able to regulate many cellular events and physiological processes (such as cell growth and differentiation, cell-cell and cell-matrix interaction, anti-infective and anti-inflammatory processes, etc.) [9-11]. On the basis of their structure and composition, GAGs are generally grouped into four main categories: 1) hyaluronic acid (HA); 2) chondroitin sulfates (CS) and dermatan sulfate (DS); (3) heparan sulfate (HS) and heparin (Hep); 4) keratan sulfate (KS).

### State of the art

The first data reported in the literature on milk GAGs are those described by Shimizu et al. [5]. Their study was not performed on whole milk but exclusively focused on milk fat globule membranes. The total GAG content was 5–10 times higher in human than in bovine milk membranes. Qualitative analyses on membranes from mature milk samples demonstrated that the major GAG in both bovine and human milk was HS (70%), with the remainder 30% represented by CS.

Further information on human milk GAG composition was later provided by the study done by Newburg et al. [6]. Even if no quantitative data were reported, it was interestingly shown that GAGs isolated from human milk were able to play an important role as anti-infective agents.

**Figure 1:**
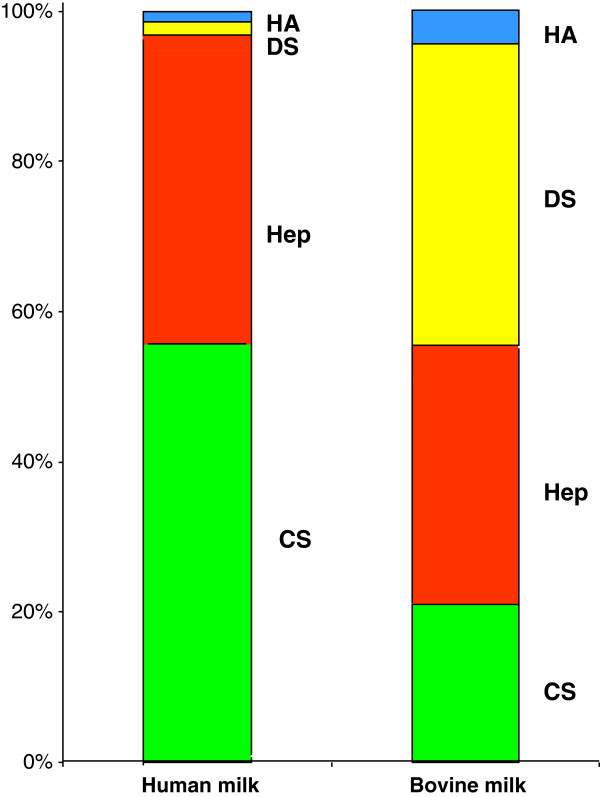
Percentage distribution of GAGs in human and bovine milk.

Recently, a complete characterization and detailed evaluation of the GAGs of human mature milk and bovine milk were performed [7]. Great differences were found between human and bovine milk in terms of both quality and quantity (Table 1) (Figure 1). The total amount of GAGs resulted about 7 times higher in human milk compared to bovine milk. Moreover on the basis of specific investigations, it was possible to precisely define the composition of the complex mixture of GAGs in human and bovine milks: CS, DS, Hep and HA were identified in both milks. The main GAGs of human milk were represented by CS (~55%) followed by Hep (~40%), whose absolute amounts were ~23 and ~7 times respectively higher than in bovine milk. On the other hand, the main bovine milk GAG was DS (~40%) followed by Hep (~30%) and CS (~21%).

**Table 1:** GAGs quantitative evaluation in human and bovine milk

	**Human milk**	**Bovine milk**
**Total GAGs *(mg/L)***	**416.2**	**60.2**
	**GAGs *(mg/L)***	**GAGs *(mg/L)***
**Chondroitin Sulfate**	**231**	**13**
**Dermatan Sulfate**	**7**	**24**
**Heparin**	**173**	**21**
**Hyaluronic acid**	**5**	**2**

**Figure 2:**
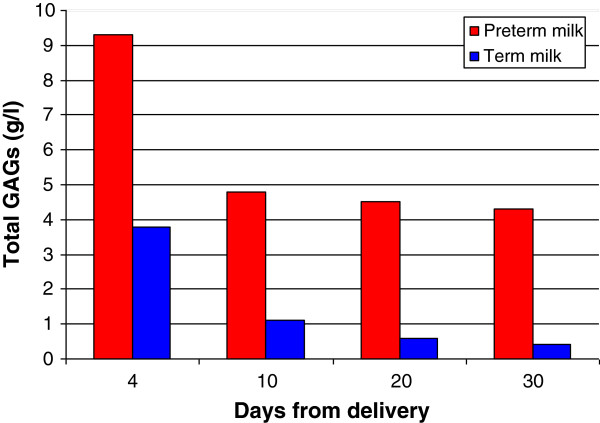
Content of GAGs in term and preterm human milk during the first month of lactation.

In a further study, the total GAG content in pooled milk from healthy mothers having delivered term or preterm newborns was evaluated during the first month of lactation [8]. In both term and preterm milk, GAGs showed a constant pattern which was essentially composed of two main polysaccharides: CS (60-70%) and Hep (30-40%). Moreover, considerable variations of GAG concentration were found during the period studied. In fact, highest values were present at 4th day (9.3 and 3.8 g/L in preterm and term milk respectively), followed by a progressive decrease up to 30th day (4.3 and 0.4 g/L) (Figure 2).

### Metabolism and potential biological functions

**Figure 3:**
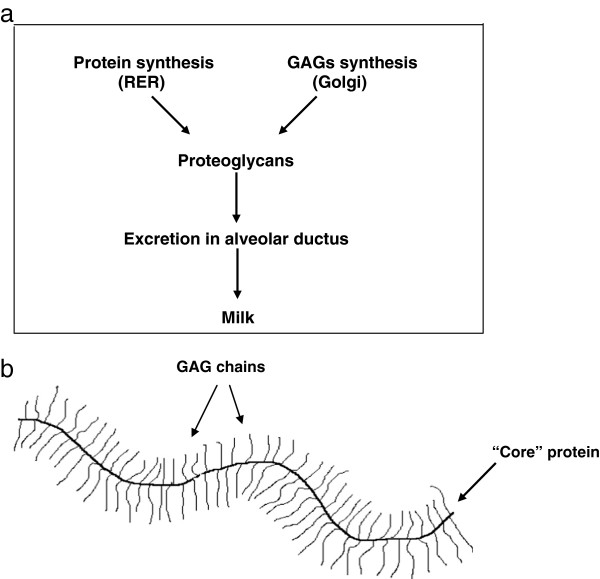
Synthesis (a) and structure (b) of a proteoglycan.

In the alveolar cells of the mammary gland, the synthetized GAG chains are first linked to a protein “core” and then excreted into the glandular ductus as the macromolecular complex of Proteoglycans (PGs) (Figure 3a and b). From the above reported results, it follows that breastfed infants ingest consistent daily amounts of GAGs (as PGs). However, at present, no data are available on their metabolic fate.

**Figure 4:**
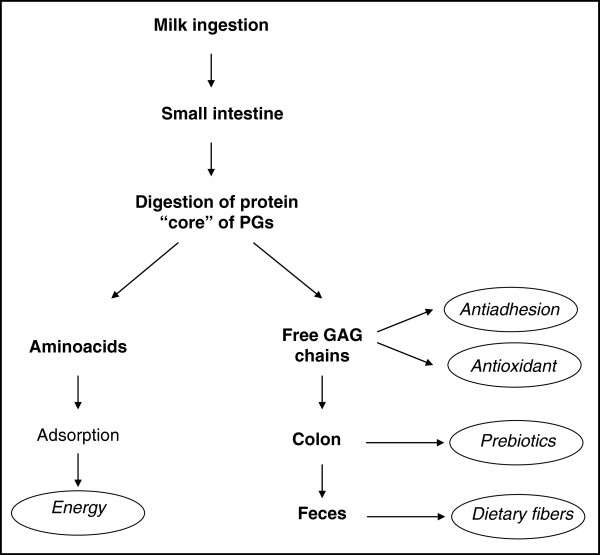
Hypothetic metabolic fate and biological functions of human milk GAGs.

It seems reasonable to hypothesize (Figure 4) that at the small intestine level the proteolytic enzymes secreted in the pancreatic juice digest the “core” protein of PGs into aminoacids, which are absorbed, resulting in the liberation of free GAG chains. As the intestinal wall and microvilli lack specific glycosidases and sulfatases, the free GAGs should persist undegraded in the upper part of the digestive system [12]. Recent studies provide further demonstration of the physiological role of GAGs in several biological processes [9,11]. In particular, it has been shown that some cell surface receptors are constituted by GAGs [11], participating directly, in this way, in the regulation of the infective processes. Therefore, human milk GAGs could have the power to interact with pathogens and to compete for their adhesion to the intestinal wall, as already demonstrated for other human milk glycans [1,2,13]. In fact, the first data available on the anti-infective role of human milk GAGs are those reported by Newburg et al. [6]. Detailed analyses performed on isolated GAGs demonstrate the presence of a complex mixture made up of DS, Hep, HS and CS. For the first time, these authors demonstrated that CS isolated from human milk was able to inhibit the binding of the HIV envelop glycoprotein gp120 to the cellular CD4 receptor. Moreover, it was recently demonstrated that HA fragments play an important role in promoting an innate antimicrobial effect in intestinal epithelial cells [14]. On the basis of these data we can suppose that the high concentration of human milk GAGs could be useful for the newborn in defence processes against several pathogens (viruses, bacteria and their toxins).

At the same level of the small intestine, the GAGs, due to their well-known properties, could contribute to the antioxidant effect of human milk, which is particularly important during the neonatal period. In fact, it has been demonstrated that CS (and other GAGs) are able to stimulate the pathway which induces the activation of antioxidant enzymes [15], and which is particularly important in preterm infants endowed with an immature defence system.

Furthermore, the undigested GAGs, reaching the colon, could behave as prebiotics, contributing to the development of bifidogenic flora as it has been demonstrated that bifidobacteria possess specific enzymes involved in the metabolism of carbohydrates [16].

Finally, a certain amount of undigested GAGs could be present in infant feces behaving as dietary fibers according to international definition.

### Future perspectives

From the review of the literature, it clearly emerges that further studies are necessary to explore the metabolic fate, the physiological role and other human milk GAGs possible positive effects on the newborn’s health. As for other glycan components such as oligosaccharides, further in vitro and in vivo studies should be performed to achieve more information on their possible antinfective, antioxidant and prebiotic effects.

Finally, new knowledge on human milk components is the starting basis for the preparation of improved infant formulas in the future.

## Abbreviations

GAGs: Glycosaminoglycans; HA: Hyaluronic acid; CS: Chondroitin sulfates; DS: Dermatan sulfate; HS: Heparan sulphate; Hep: Heparin; KS: Keratan sulphate; PGs: Proteoglycans.

## Competing interests

The authors declare that they have no competing interest.

## Authors’ contributions

All authors read and approved the final manuscript.
